# Supplementary material for: The companion of cellulose synthase 1 confers salt tolerance through a Tau-like mechanism in plants
Source: Nat Commun. 2019 Feb 20;10:857. doi: 10.1038/s41467-019-08780-3 (PMC6382854; doi:10.1038/s41467-019-08780-3)
Supplement: Supplementary file 1 — Supplementary Information [file 41467_2019_8780_MOESM1_ESM.pdf]

Supplementary Information

''The Companion of Cellulose Synthase 1 confers salt tolerance through a Tau-like mechanism in plants”

Christopher Kesten, Arndt Wallmann et al

a

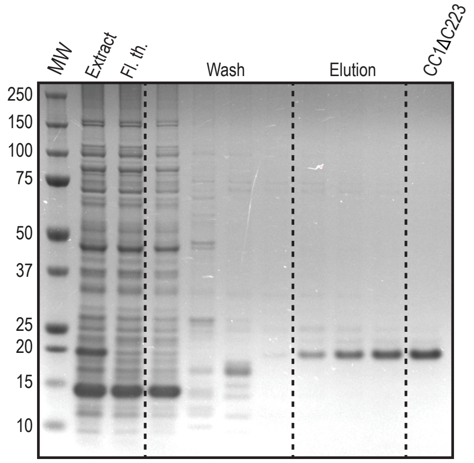
MW
Extract
Fl. th.
Wash
Elution
CC1ΔC223
250
150
100
75
50
37
25
20
15
10

b

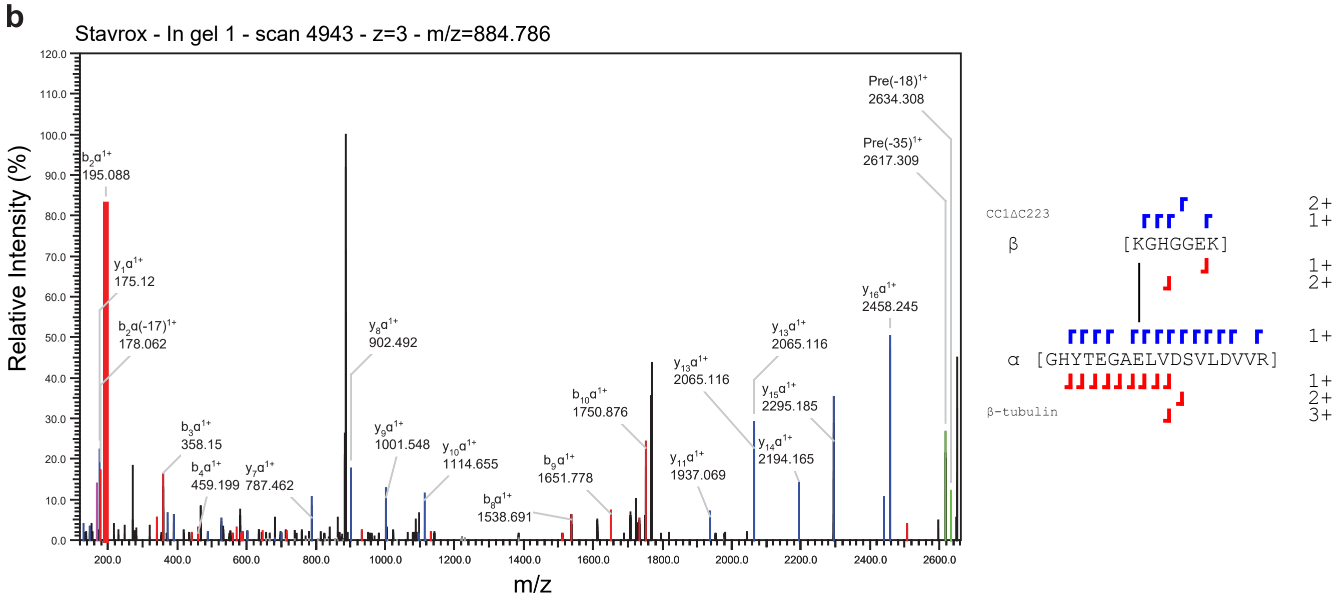
Stavrox - In gel 1 - scan 4943 - z=3 - m/z=884.786
Relative Intensity (%)
m/z
Pre(-18)1+ 2634.308
Pre(-35)1+ 2617.309
b2α1+ 195.088
y1α1+ 175.12
b2α(-17)1+ 178.062
y8α1+ 902.492
y16α1+ 2458.245
y13α1+ 2065.116
y13α1+ 2065.116
y15α1+ 2295.185
y14α1+ 2194.165
b10α1+ 1750.876
y11α1+ 1937.069
b9α1+ 1651.778
b8α1+ 1538.691
y10α1+ 1114.655
y9α1+ 1001.548
b3α1+ 358.15
b4α1+ 459.199
y7α1+ 787.462
CC1ΔC223
β [KGHGGEK]
α [GHYTEGAELVDSVLDVVR]
β-tubulin
2+
1+
1+
2+
1+
1+
2+
3+

c

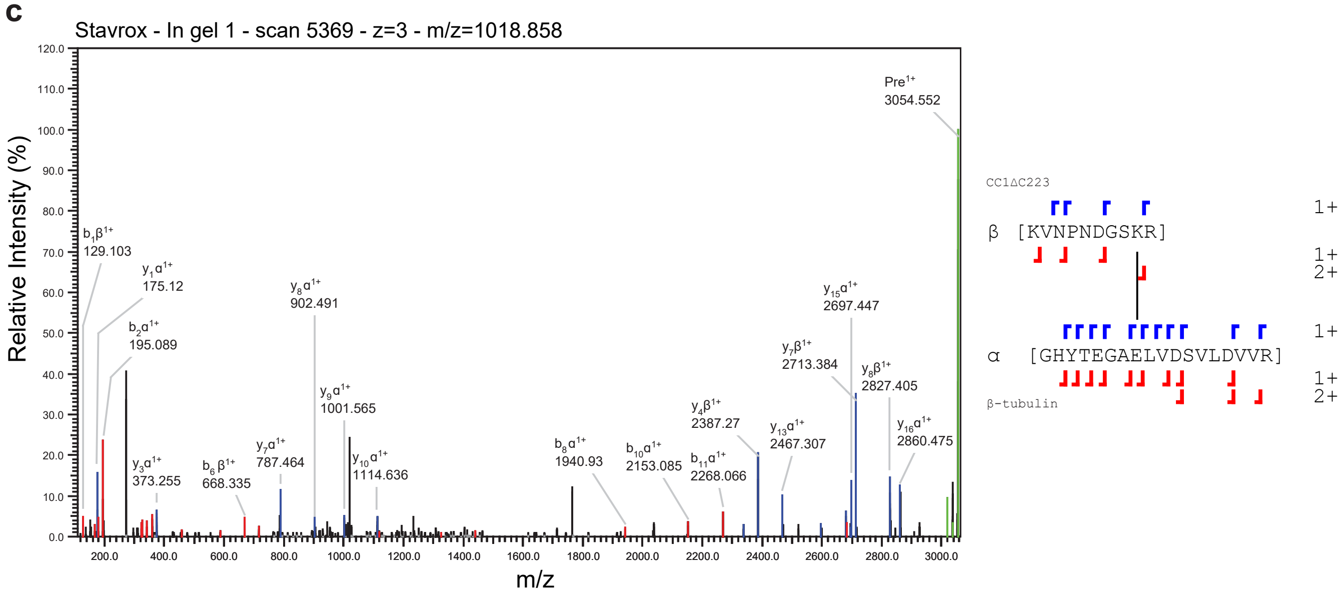
Stavrox - In gel 1 - scan 5369 - z=3 - m/z=1018.858
Relative Intensity (%)
m/z
Pre1+ 3054.552
b1β1+ 129.103
y1α1+ 175.12
b2α1+ 195.089
y8α1+ 902.491
y15α1+ 2697.447
y7β1+ 2713.384
y8β1+ 2827.405
y4β1+ 2387.27
y13α1+ 2467.307
y16α1+ 2860.475
y9α1+ 1001.565
y3α1+ 373.255
b6β1+ 668.335
y7α1+ 787.464
y10α1+ 1114.636
b8α1+ 1940.93
b10α1+ 2153.085
b11α1+ 2268.066
CC1ΔC223
β [KVNPNDGSKR]
α [GHYTEGAELVDSVLDVVR]
β-tubulin
1+
1+
2+
1+
1+
2+

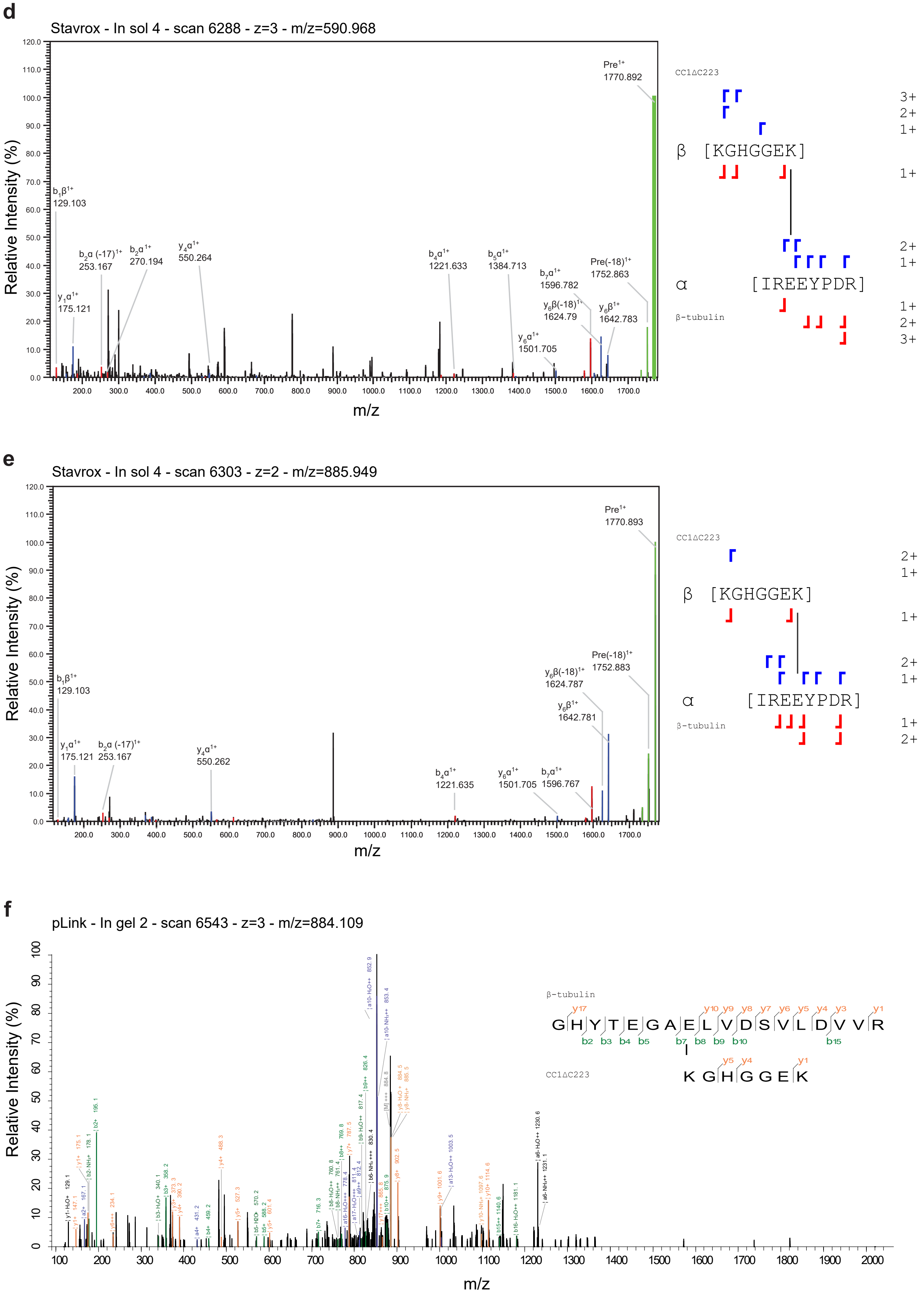

d
Stavrox - In sol 4 - scan 6288 - z=3 - m/z=590.968
Relative Intensity (%)
m/z
Pre1+ 1770.892
b1β1+ 129.103
y1α1+ 175.121
b2α (-17)1+ 253.167
b2α1+ 270.194
y4α1+ 550.264
b4α1+ 1221.633
b5α1+ 1384.713
y6α1+ 1501.705
b7α1+ 1596.782
y6β(-18)1+ 1624.79
y6β1+ 1642.783
Pre(-18)1+ 1752.863
CC1ΔC223
β [KGHGGEK]
α [IREEYPDR]
β-tubulin
3+
2+
1+
e
Stavrox - In sol 4 - scan 6303 - z=2 - m/z=885.949
Relative Intensity (%)
m/z
Pre1+ 1770.893
b1β1+ 129.103
y1α1+ 175.121
b2α (-17)1+ 253.167
y4α1+ 550.262
b4α1+ 1221.635
y6α1+ 1501.705
b7α1+ 1596.767
y6β(-18)1+ 1624.787
y6β1+ 1642.781
Pre(-18)1+ 1752.883
CC1ΔC223
β [KGHGGEK]
α [IREEYPDR]
β-tubulin
f
pLink - In gel 2 - scan 6543 - z=3 - m/z=884.109
Relative Intensity (%)
m/z
β-tubulin
GHYTEGAELVDSVLDVVR
CC1ΔC223
KGHGGEK

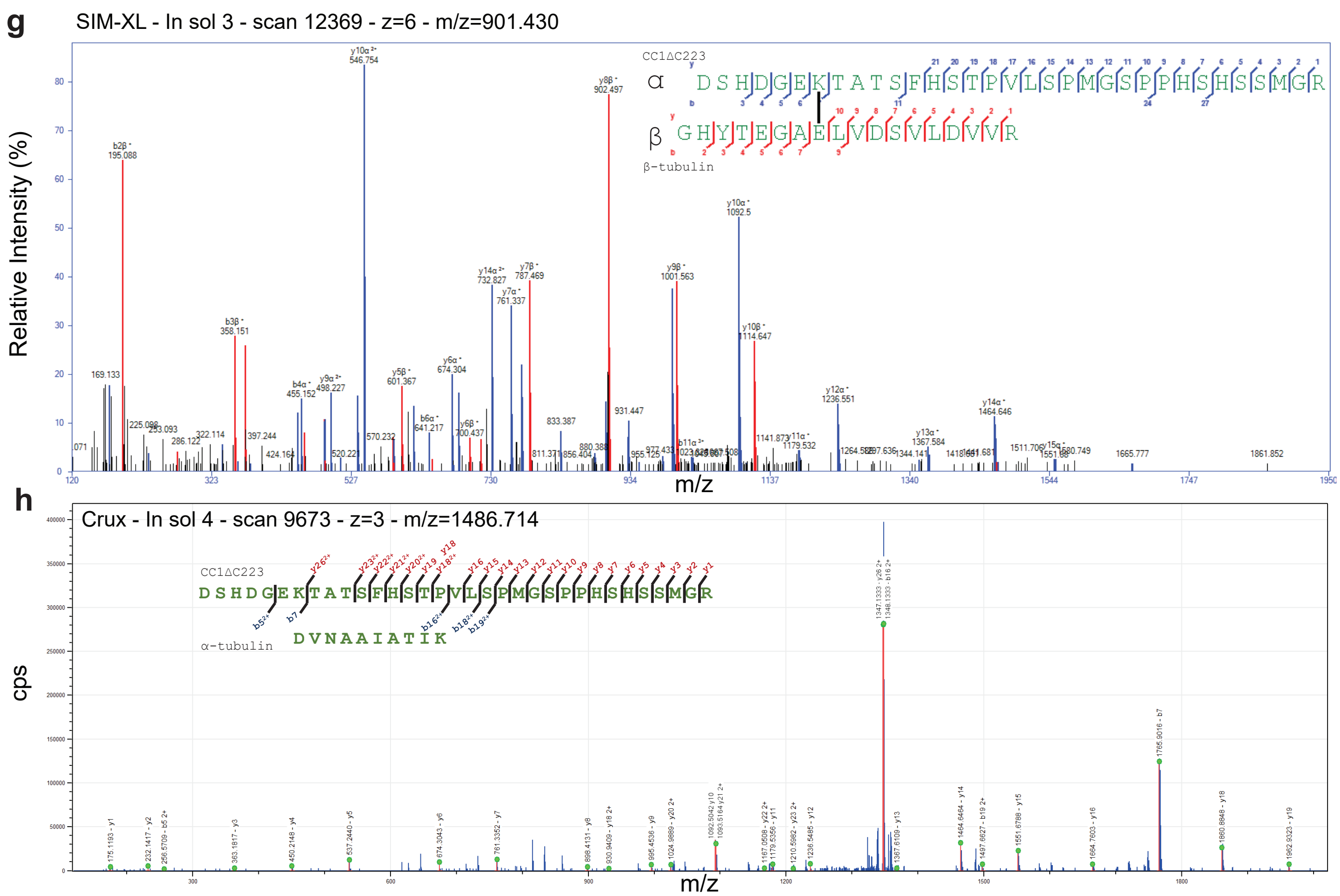

Supplementary Figure 1. The N-terminus of CC1 binds to residues of both α-Tubulin and β-Tubulin

a SDS-Page showing the purification of 6xHis-CC1ΔC223 using nickel chelate chromatography with 50 mM sodium phosphate buffer (pH 7.0) and 200 mM NaCl for the cross-linking reactions as depicted in Figure 1a. MW = molecular weight marker, Fl. th. = flow through.

b Annotated MS2 spectra (b-h) representing the identified cross-links between CC1ΔC223 and tubulin; Representative annotated spectra are shown for each cross-link; File defines the filename of the datafile available at PRIDE (PXD009260); In gel/In sol defines the digestion method of proteins; Ion series and identified peptide composition are shown to the right (b-e) or in front of MS2 spectrum (f-h); see also Table S2. Spectrum identified by Stavrox, pLink, SIM-XL and Crux; annotated with Stavrox; File: In gel 1; Scan 4943; z=3; m/z=884.786. Blue peaks represent y-ions, red peaks represent b-ions. Only major peaks are labeled.

c Spectrum identified by Stavrox; annotated with Stavrox; File: In gel 1; Scan 5369; z=3; m/z=1018.858. Blue peaks represent y-ions, red peaks represent b-ions. Only major peaks are labeled.

d Spectrum identified by Stavrox and pLink; annotated with Stavrox; File: In sol 4; Scan 6288; z=3; m/z=590.968. Blue peaks represent y-ions, red peaks represent b-ions. Only major peaks are labeled.

e Spectrum identified by Stavrox and pLink; annotated with Stavrox; File: In sol 4; Scan 6303; z=2; m/z=885.949. Blue peaks represent y-ions, red peaks represent b-ions. Only major peaks are labeled.

f Spectrum identified by pLink and Crux; annotated with pLink (pLabel); File: In gel 2; Scan 6543; z=3; m/z=884.109. Orange peaks represent y-ions, green peaks represent b-ions blue peaks represent a-ions.

g Spectrum identified by SIM-Xl; annotated with SIM-XL; File: In sol 3; Scan 12369; z=6; m/z=901.430. Blue peaks represent y-and b-ions belonging to CC1ΔC223, red peaks represent y- and b-ions belonging to β-tubulin. Only major peaks are labeled.

h Spectrum identified by Crux; annotated with mMass; File: In sol 4; Scan 9673; z=3; m/z=1486.714. Red peaks represent y-ions, blue peaks represent b-ions.

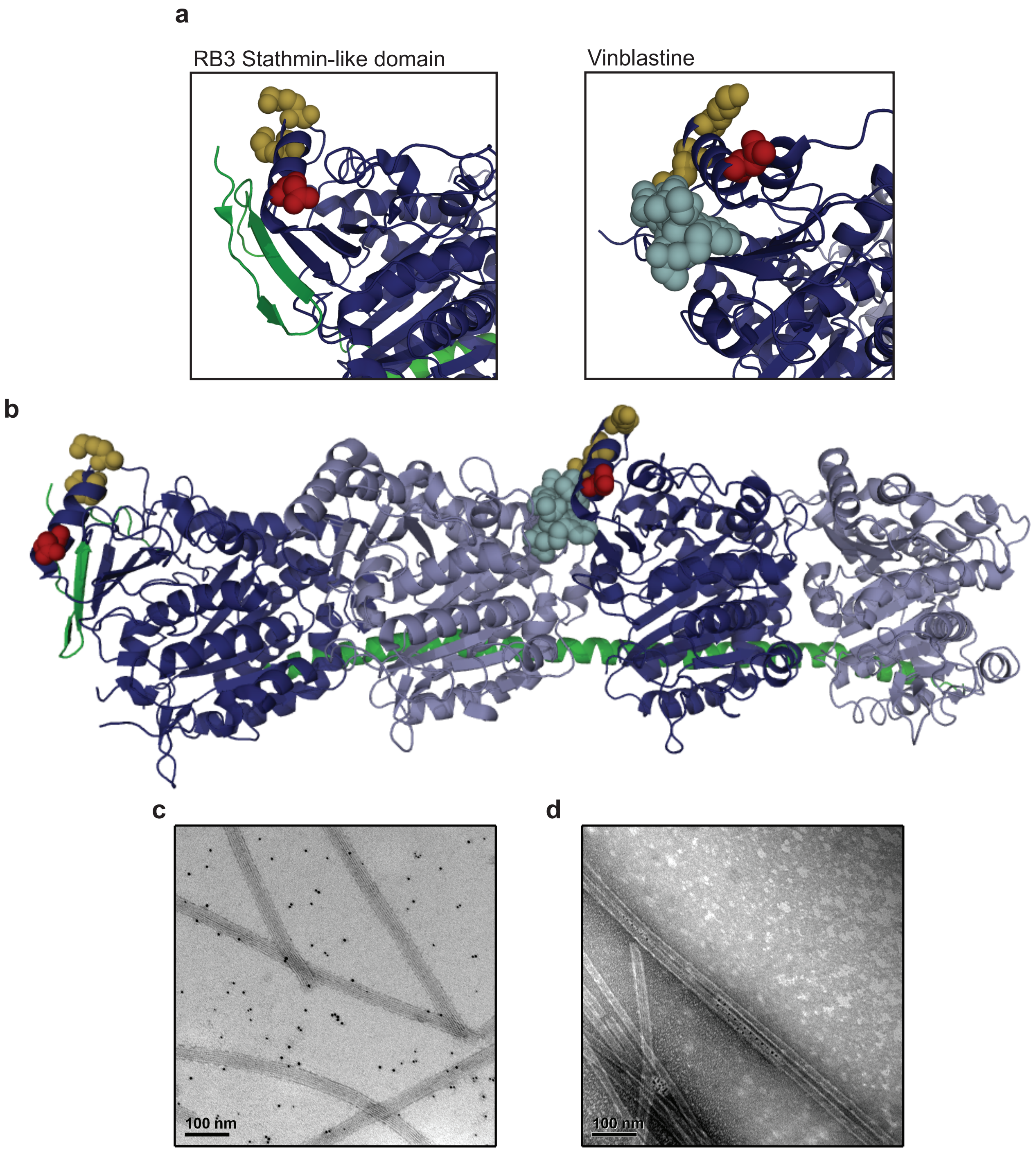

Supplementary Figure 2. The N-terminus of CC1 binds to residues on α-Tubulin that are in close vicinity to Tau-microtubule interaction sites and cross-links microtubules

a-b Comparison of CC1ΔC223 cross-link position (red) with Tau cross-links (yellow) at the α-tubulin dimer interface (dark blue). The cross-link position is in close vicinity to the Vinblastine (cyan) and RB3 SLD binding site (PDB code: 4EB6 [http://dx.doi.org/10.2210/pdb4EB6/pdb]).

c Nano-gold distribution when BSA is used as a control. Note that the gold labeling is now speckled over the whole image and is not specifically found to decorate microtubules. Scale bar = 100 nm.

d CC1ΔC223 decoration when more than two microtubules are in close vicinity to each other, i.e. one microtubule can become linked to multiple microtubules *via* CC1ΔC223. Scale bar = 100 nm.

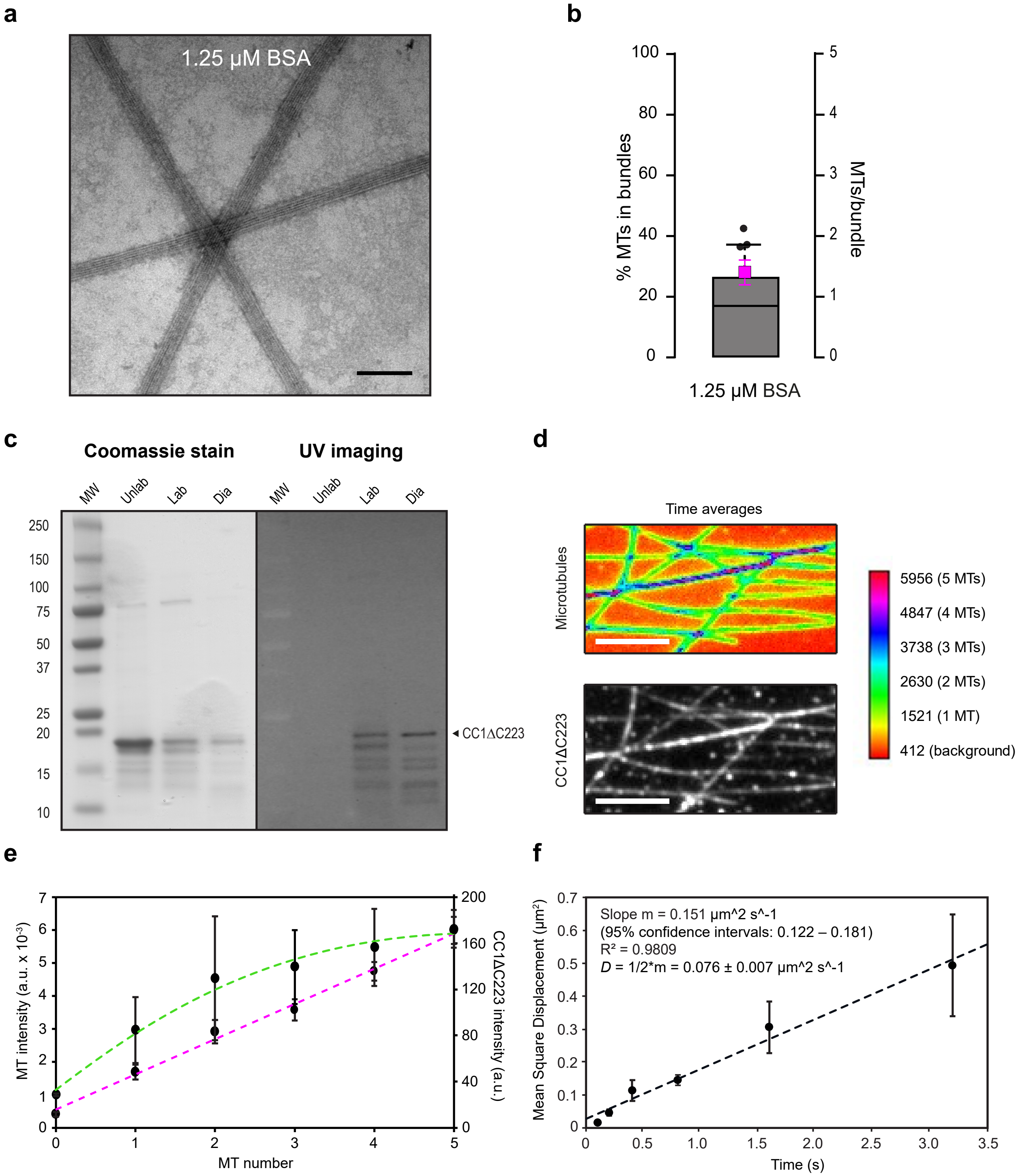

Supplementary Figure 3. The N-terminus of CC1 can diffuse along the microtubule lattice

a Transmission electron microscopy (TEM) of negatively-stained taxol-stabilized microtubules after addition of BSA as a negative control during microtubule polymerization. Scale bar = 100 nm.

b Quantification of the proportion of microtubules in bundles (left y-axis, box plots: Centerlines show the medians; box limits indicate the 25th and 75th percentiles; whiskers extend to the 10th and 90th percentiles, outliers are represented by dots) and number of microtubules/bundle (right y-axis, magenta data point: mean +/- SEM) with BSA (quantified from images such as those in (**a**)).

Continued on next page.

c SDS-PAGE of CF488A labeling of 6xHis-CC1ΔC223. Left panel shows coomassie stain of the SDS-PAGE, right panel shows the same gel before coomassie staining under UV light. Unlab = 6xHis-CC1ΔC223 before labeling; Lab = 6xHis-CC1ΔC223 after labeling reaction and nickel chelate purification; Dia = final CF488A-labeled 6xHis-CC1ΔC223 after dialysis and concentration.

d False-colored microtubules allow counting of the number of microtubules in a bundle (top). Time-average image of CC1ΔC223 foci (bottom) shows more fluorescence on bundled microtubules. Scale bar = 5 µm.

e Microtubule bundle intensity scales linearly with number of microtubules (mean +/- SD, n = 20, 16, 9, 14, and 2 filaments; regression (dotted magenta line) = 1109 * x + 412; R^2 = 0.99283, intensity averaged over 50 s). CC1ΔC223 intensity scales with microtubule numbers in bundles (dotted green line) (mean +/- SD).

f Mean squared displacement (MSD) of CC1ΔC223 plotted against the time increment Δt, in which the displacement was measured, error bars show SEM, D = diffusion coefficient. Assuming one-dimensional diffusion along microtubules (MSD = 2D*Δt) we obtained a diffusion constant of 0.076 +/- 0.007 µm^2 s^-1 (mean +/- SD, N = 6 time increments with n = 830, 370, 220, 187, 77, and 31 displacements from 50 molecules).

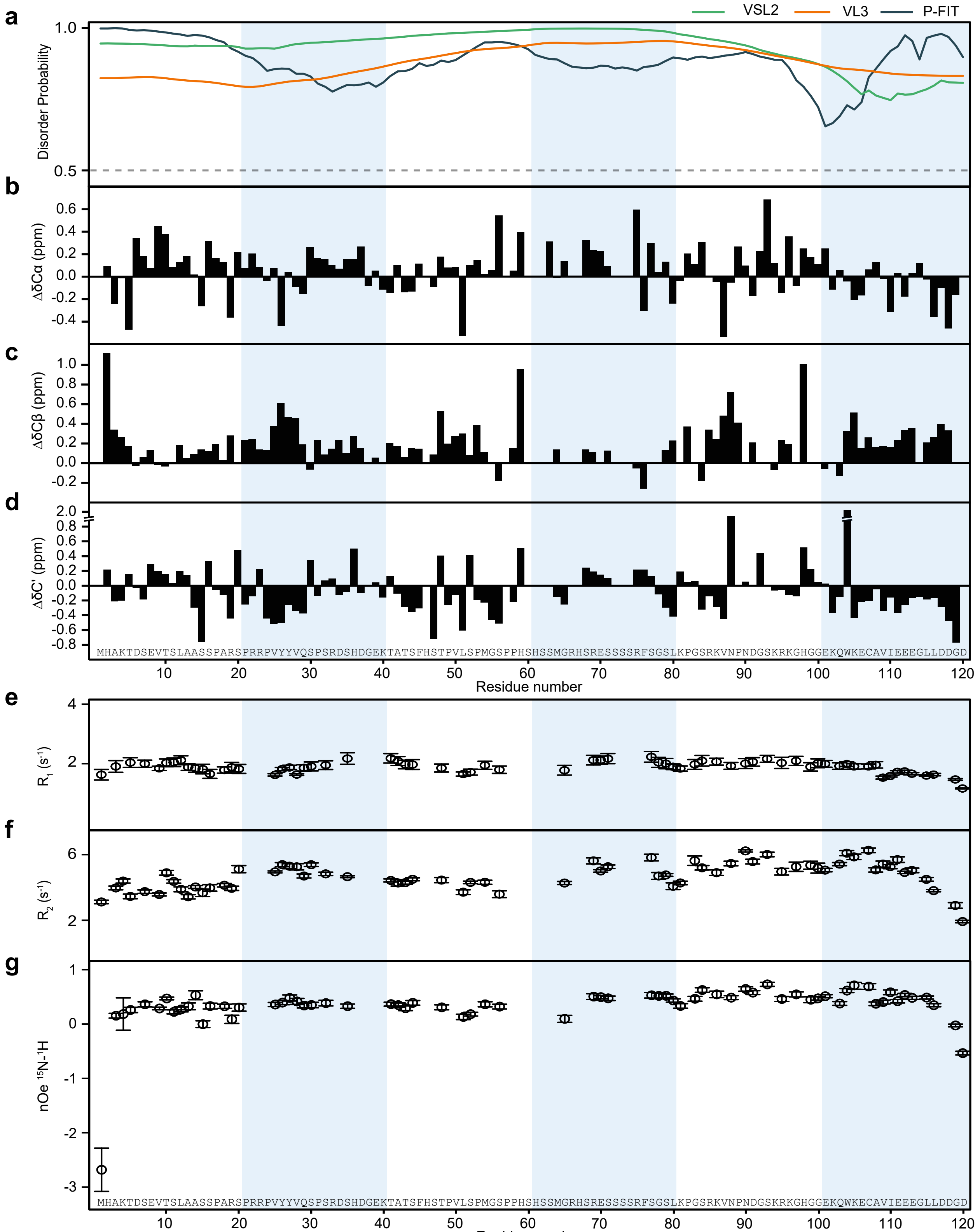

Supplementary Figure 4. Structure prediction and dynamics of CC1ΔC223

a Prediction of the degree of disorder of CC1ΔC223 with the algorithms VSL2 (green), VL3 (orange) and P-FIT (black). Regions with a disorder probability above 0.5 are considered disordered.

b-d ΔδCα, ΔδCβ and ΔδC' values for CC1ΔC223. Experimental Cα, Cβ and C' chemical shifts were subtracted from the respective random coil values for each amino acid type from the random-coil chemical shift library ncIDP.

e-f Residue-resolved ^{15}N longitudinal (R_1) and transverse (R_2) relaxation rates of CC1ΔC223 in solution. Error bars, SD.

g ^{15}N–^{1}H hetero-nuclear Overhauser effect (NOE) data of CC1ΔC223 in solution. Error bars, SD.

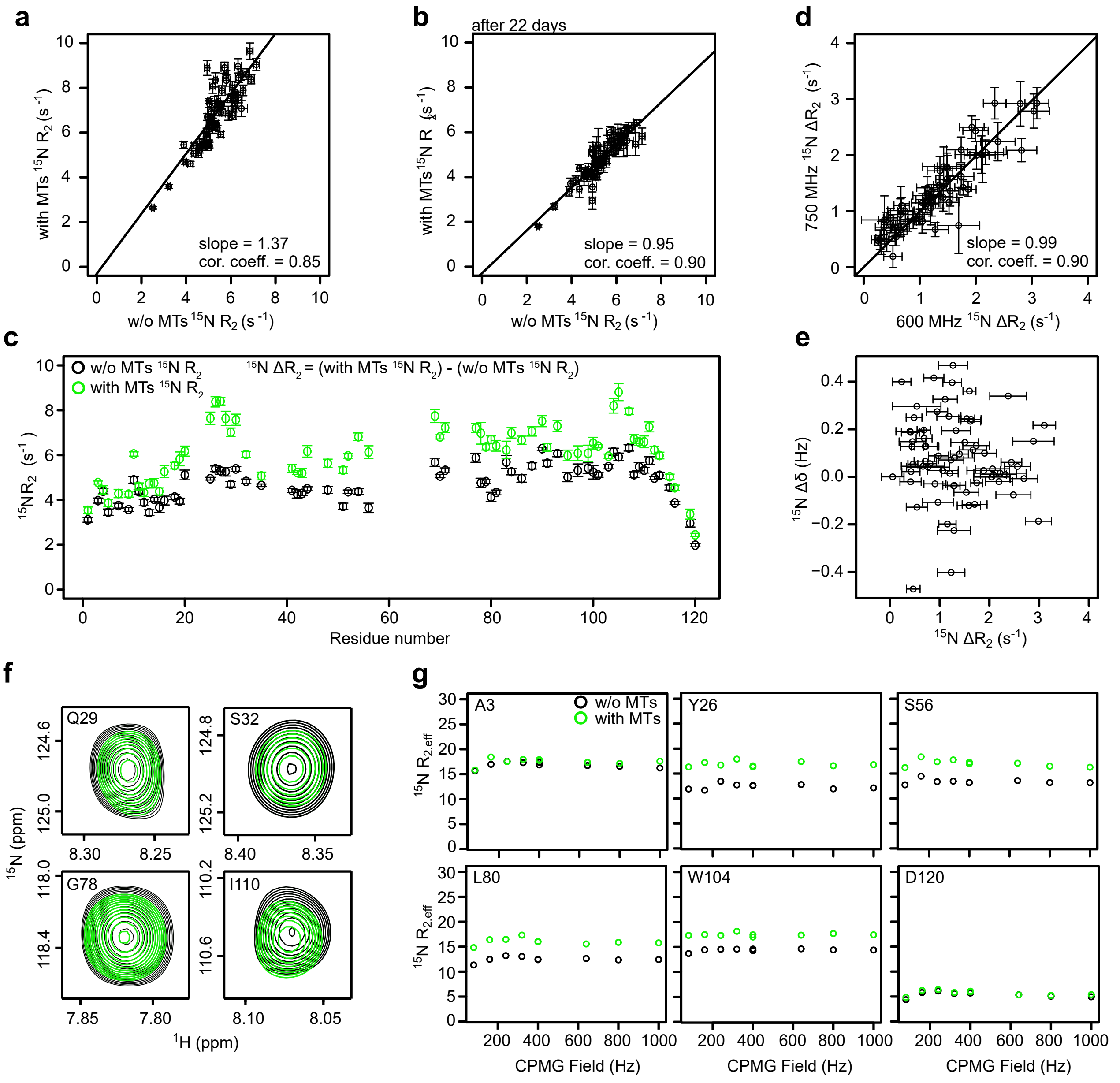

Supplementary Figure 5. Changes in relaxation rates and chemical shifts for the CC1 N-terminus in the presence of microtubules

a CC1ΔC223 ^{15}N-R_2 relaxation rates before and after complexation with microtubules. Error bars, SD.

b Comparison of the CC1ΔC223 ^{15}N-R_2 relaxation of the free form after the microtubules disintegrated. Error bars, SD.

c Residue specific ^{15}N-R_2 relaxation rates at 750 MHz before (black) and after (green) addition of microtubules. The observed changes in the relaxation rate are defined as ^{15}N-ΔR_2. Error bars, SD.

d Correlation of ^{15}N-ΔR_2 values recorded at spectrometer frequencies of 600 and 750 MHz. ^{15}N-ΔR_2 values do not depend on magnetic field. Error bars, SD.

e ^{15}N-ΔR_2 values do not correlate with ^{15}N exchange-induced chemical shifts that occur upon complex formation. Error bars, SD.

f Selection of peaks of the ^{1}H-^{15}N HSQC spectra of CC1ΔC223 in the absence (black) and the presence (green) of microtubules.

g Examples of ^{15}N CPMG relaxation dispersion curves for free CC1ΔC223 (black) and in the presence of microtubules (green) at 600 MHz.

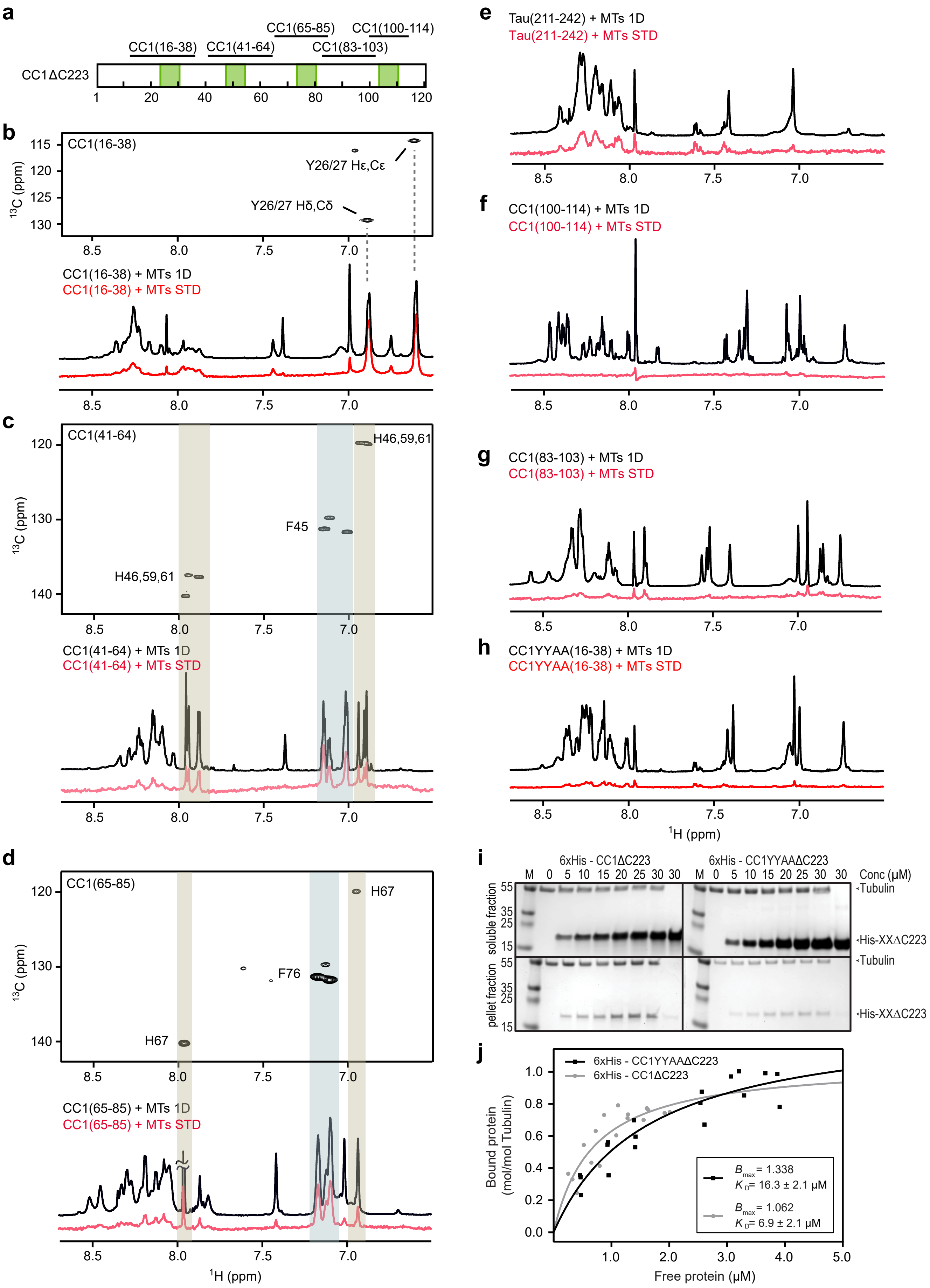

a
CC1(16-38)
CC1(41-64)
CC1(65-85)
CC1(83-103)
CC1(100-114)
CC1ΔC223
b
CC1(16-38)
Y26/27 Hε,Cε
Y26/27 Hδ,Cδ
CC1(16-38) + MTs 1D
CC1(16-38) + MTs STD
c
CC1(41-64)
H46,59,61
F45
CC1(41-64) + MTs 1D
CC1(41-64) + MTs STD
d
CC1(65-85)
H67
F76
CC1(65-85) + MTs 1D
CC1(65-85) + MTs STD
^{13}C (ppm)
^{1}H (ppm)
e
Tau(211-242) + MTs 1D
Tau(211-242) + MTs STD
f
CC1(100-114) + MTs 1D
CC1(100-114) + MTs STD
g
CC1(83-103) + MTs 1D
CC1(83-103) + MTs STD
h
CC1YYAA(16-38) + MTs 1D
CC1YYAA(16-38) + MTs STD
i
6xHis - CC1ΔC223
6xHis - CC1YYAAΔC223
Conc (μM)
Tubulin
His-XXΔC223
soluble fraction
pellet fraction
j
6xHis - CC1YYAAΔC223
6xHis - CC1ΔC223
B_{max} = 1.338
K_D = 16.3 ± 2.1 μM
B_{max} = 1.062
K_D = 6.9 ± 2.1 μM
Bound protein (mol/mol Tubulin)
Free protein (μM)

Supplementary Figure 6. Binding of peptides derived from the CC1 N-terminus to microtubules

a Cartoon-overview of CC1ΔC223. The location of the microtubule-binding regions is highlighted in green and the peptides used for saturation transfer difference (STD) NMR are indicated.

b-**h** Comparison of one-dimensional ^{1}H spectra (black) and STD-NMR spectra (red) of CC1(16-38) (**b**) CC1(41-64) (**c**), CC1(65-85) (**d**) Tau(211-242) (**e**), CC1(100-114) (**f**), CC1(83-103) (**g**) and the mutated CC1YYAA(16-38) peptide (**h**). The data of CC1(16-38) (**b**), CC1(41-64) (**c**) and CC1(65-85) (**d**) are shown in combination with two-dimensional ^{1}H-^{13}C HMQC spectra. In these peptides, STD signals were detected and assigned to the aromatic side chains of tyrosine, histidine and phenylalanine residues in the respective regions. The positive control peptide Tau(211-242) containing the N-terminal microtubule binding region from Tau [22] showed significant intensities in the amide region. The negative control peptide CC1(83-103) representing the most prominent linker region of the protein and CC1(100-114) showed no binding in the present conditions.

i Typical coomassie gels used for the estimation of the microtubule dissociation constant for 6xHis-CC1ΔC223 and 6xHis-CC1YYAAΔC223. Molecular size of tubulin and 6xHis-CC1ΔC223/6xHis-CC1YYAAΔC223 (His-XXΔC223) are indicated by arrowheads.

j The microtubule binding constants of 6xHis-CC1ΔC223 (6.9 ± 2.1 µM) and 6xHis-CC1YYAAΔC223 (16.3 ± 4.1 µM, Best-fit values ± Std. Error) were determined by estimation of binding of the respective protein when microtubule levels were constant. K_D was calculated by fitting a saturation binding curve onto the data; N = 3 independent experiments.

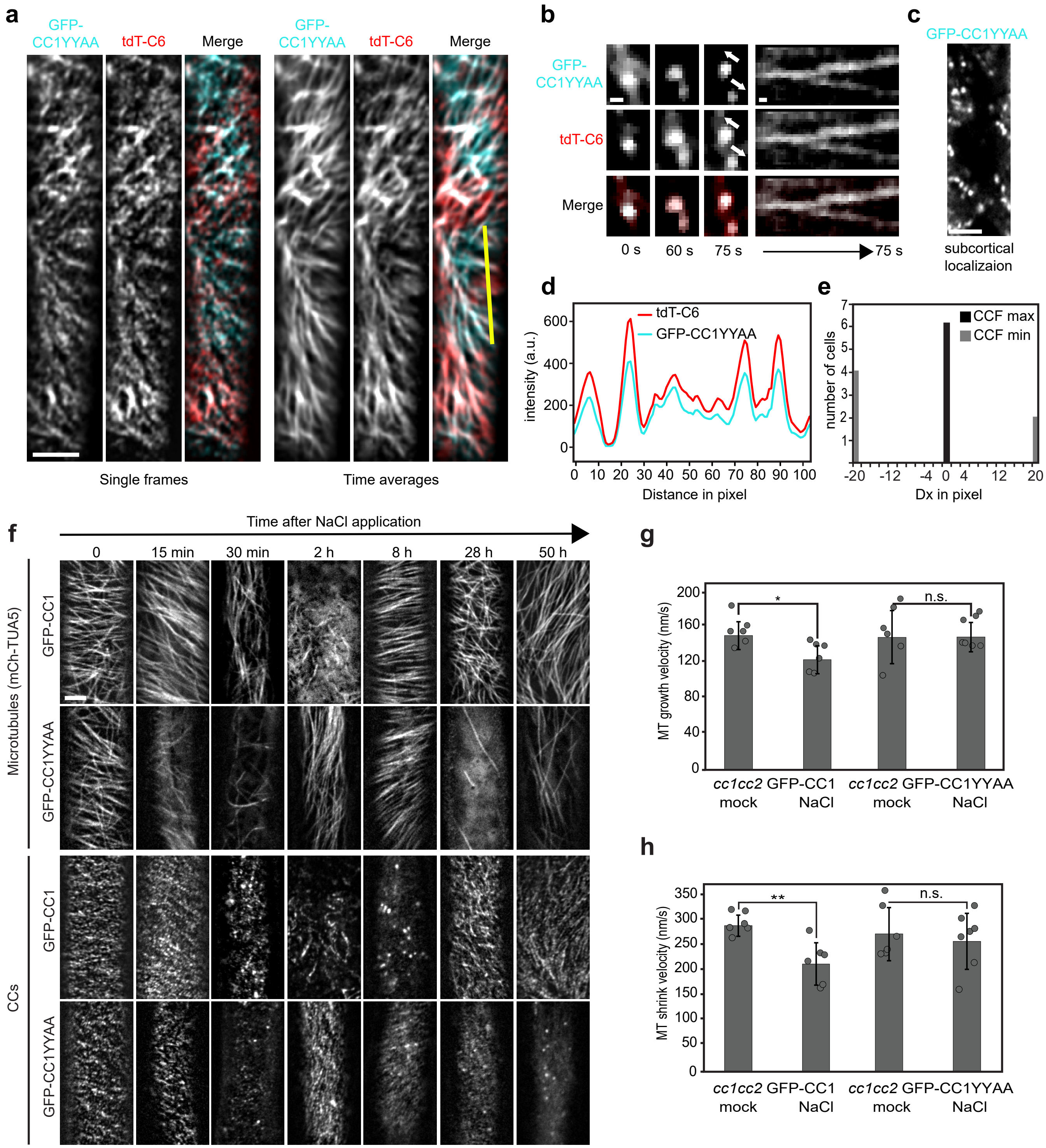

Supplementary Figure 7. CC1 and microtubule behavior upon salt exposure

a GFP-CC1YYAA and tdTomato-CesA6 (tdT-C6) co-localize and co-migrate as foci at the plasma membrane. Scale bar = 5 µm.

b Left panel; Time frames of individual plasma membrane–located fluorescent GFP-CC1YYAA and tdT-C6 foci reveal that they get delivered to the plasma membrane as one (at time 0 s). The foci split into two foci in both channels that migrate bi-directionally (time 60 and 75 s). Right panel; Kymographs of 75 s movies of GFP-CC1YYAA and tdT-C6 expressing cells from frames in left panel. Scale bars = 0.5 µm.

c Subcortical localization of GFP-CC1YYAA. The localization is reminiscent of the trans-Golgi network (see Endler et al., 2015 for details). Scale bar = 5 µm.

d Intensity plot of GFP-CC1YYAA and tdT-C6 from transect in (**a**) along the depicted yellow line.

Continued on next page.

e Quantification of GFP-CC1YYAA and tdT-C6 co-localization in hypocotyl cells of three-day-old *cc1cc2* etiolated seedlings. Cross- correlation coefficients (CCFs) were calculated at dx = 0 and shifted images with dx ± 20 pixels using the van Steensel's algorithm. Note the highest CCFs (CCF max, black bar) were detected at dx = 0 for n = 6 cells. Gray bars represent the shifts (dx) that lead to the lowest CCF (CCF min), where overlap of green and red signals was lowest.
f Typical microtubule and GFP-CC (GFP-CC1 or GFP-CC1YYAA) coverage at the cell cortex and plasma membrane, respectively, in 3-day-old mCh-TUA5 and GFP-CC1 or GFP-CC1YYAA expressing *cc1cc2* etiolated seedlings after exposure to 200 mM NaCl for indicated times. T = 0 indicates time just prior to salt exposure. Scale bar = 5 µm.
g-**h**. Microtubule (MT) growth (**g**) and shrink (**h**) velocities in *cc1cc2* GFP-CC1 and *cc1cc2* GFP-CC1YYAA cells. The dynamics were estimated using a custom-made ImageJ macro (see Methods section) and were done on cells 2 h after salt stress (*cc1cc2* GFP-CC1YYAA mutant cells) and 6 h after salt stress (*cc1cc2* GFP-CC1) in twelve cells from twelve different seedlings. These time points were chosen based on microtubule array re-establishment timing as indicated in **f** and Figure 5g. Dots represent individual data points of the corresponding bars. Values are mean +/- SD, Welch's unpaired *t*-test; * p-value ≤ 0.05; ** p-value ≤ 0.01; n.s., no significant difference.

Supplementary Table 1: Primers used in the manuscript (5’-3’)

No.	Name	Sequence (5’-3’)
1	cc1 LP	TTAATGACGTACGCGTTTGTG
2	cc1 RP	GAAGACTTACGGATCCGGAAC
3	LB_SAIL	TGACGCCATTTCGCCTTTTC
4	cc2 LP	CAGATCACTATTGGCTCTGGCTC
5	cc2 RP	AAATAGATGTGACAGTGCAGTTATTCG
6	cc2 insert fw	ATGCACGCGAAGACCGACTCCG
7	08409_GABI Insert	ATATTGACCATCATACTCATTGC
8	GFP fw	ATGGTGAGCAAGGGCGAGG
9	CC1 YYtoAA rev	TGGACTGCGGCGACTGG
10	mut general fw	GTTGTAAAACGACGGCCAGTC
11	YY to AA rev	TGGTGATTGGACTGCGGCGACTGGTCTACGTGGAGAT CTGGCTGG
12	YY to AA fw	CCAGTCGCCGCAGTCCAATCACCATCACGCGACTCTC ACGACG
13	mut general rev	CTCTTCCTTCCCTGATAAAATTTCTTAAC
14	CC1_3C fw	CTGGAAGTTCTGTTCCAGGGTCCGATGCACGCCAAAA CCGATTCCGAGG
15	CC1_3C rev	CGGACCCTGGAACAGAACTTCCAGCATGGGGTGATGG TGATGGTGATGTTTC
16	CC1_3C_YYAA fw	CGTAGACCAGTCGCCGCAGTCCAATCACCATCACGCG ACTCTCACGACGG
17	CC1_3C_YYAA rev	GGTGATTGGACTGCGGCGACTGGTCTACGTGGAGATC TGGCTGGTGATGATGC

Supplementary Table 2. Identified cross-links between 6xHis-CC1ΔC223 and either α-(TBA1A_PIG) or β-tubulin (TBB_PIG) using EDC

App/ digestion	File	scan No.	z	m/z	M+H+	Identified peptide	Protein 1	Protein 2	link site 1 (-His-tag)	link site 2
**Stavrox**										
in gel	In gel 1	5369	3	1018.858	3054.559	KVNPNDGSKR, GHYTEGAELVDSVLDVVR	6xHis-CC1 ΔC223	TBB_PIG	K122 (K94)	E111
in gel	In gel 1	4943	3	884.786	2652.344	KGHGGEK, GHYTEGAELVDSVLDVVR	6xHis-CC1 ΔC223	TBB_PIG	K124 (K96)	E111
in sol	In sol 3	6371	2	885.948	1770.889	KGHGGEK,IREEYPDR	6xHis-CC1 ΔC223	TBB_PIG	K124 (K96)	E158
in sol	In sol 4	6281	2	885.949	1770.890	KGHGGEK,IREEYPDR	6xHis-CC1 ΔC223	TBB_PIG	K124 (K96)	E158
in sol	In sol 4	6303	2	885.949	1770.891	KGHGGEK,IREEYPDR	6xHis-CC1 ΔC223	TBB_PIG	K124 (K96)	E158
in sol	In sol 4	6288	3	590.968	1770.889	KGHGGEK,IREEYPDR	6xHis-CC1 ΔC223	TBB_PIG	K124 (K96)	E158
**pLink**										
in gel	In gel 1	4943	3	884.115	2652.344	KGHGGEK, GHYTEGAELVDSVLDVVR	6xHis-CC1 ΔC223	TBB_PIG	K124 (K96)	E111
in gel	In gel 2	6543	3	884.109	2652.328	KGHGGEK, GHYTEGAELVDSVLDVVR	6xHis-CC1 ΔC223	TBB_PIG	K124 (K96)	E111
in sol	In sol 3	6371	2	885.445	1770.890	KGHGGEK,IREEYPDR	6xHis-CC1 ΔC223	TBB_PIG	K124 (K96)	E158
in sol	In sol 4	6281	2	885.445	1770.890	KGHGGEK,IREEYPDR	6xHis-CC1 ΔC223	TBB_PIG	K124 (K96)	E158
in sol	In sol 4	6303	2	885.445	1770.891	KGHGGEK,IREEYPDR	6xHis-CC1 ΔC223	TBB_PIG	K124 (K96)	E158
in sol	In sol 4	6288	3	590.296	1770.889	KGHGGEK,IREEYPDR	6xHis-CC1 ΔC223	TBB_PIG	K124 (K96)	E158
**SIM-XL**										
in gel	In gel 1	4943	3	884.786	2651.330	KGHGGEK, GHYTEGAELVDSVLDVVR	6xHis-CC1 ΔC223	TBB_PIG	K124 (K96)	E111
in sol	In sol 3	12369	6	901.430	5402.580	DSHDGEKTATSFHSTPVLS PMGSPPHSHSSMGR, GHYTEGAELVDSVLDVVR	6xHis-CC1 ΔC223	TBB_PIG	K68 (K40)	E111
in sol	In sol 4	12405	6	901.430	5402.580	DSHDGEKTATSFHSTPVLS PMGSPPHSHSSMGR, GHYTEGAELVDSVLDVVR	6xHis-CC1 ΔC223	TBB_PIG	K68 (K40)	E111
**Crux**										
in gel	In gel 1	4943	3	884.786	2652.344	KGHGGEK, GHYTEGAELVDSVLDVVR	6xHis-CC1 ΔC223	TBB_PIG	K124 (K96)	E111
in gel	In gel 2	6543	3	884.781	2652.328	KGHGGEK, GHYTEGAELVDSVLDVVR	6xHis-CC1 ΔC223	TBB_PIG	K124 (K96)	E111
in sol	In sol 4	9673	3	1486.714	4458.126	DSHDGEKTATSFHSTPVLS PMGSPPHSHSSMGR, DVNAAIATIK	6xHis-CC1 ΔC223	TBA1A_ PIG	K68 (K40)	D327

A total of 4,346 potential intra-protein cross-links (cumulative) and 160 potential inter-proteins cross-links were found. Extensive manual curation led to the positively identified peptides below. Peptides are sorted by program (e.g. Stavrox) and digestion method of cross-linked proteins (in gel or in solution). Identified (linked) peptides from the two linked proteins are separated by a comma. Tubulin alpha-1A chain and tubulin beta chain from pig are abbreviated with their UniProt IDs (TBA1A_PIG and TBB_PIG). File defines the file name of the mass spec raw data available in PRIDE (PXD009260) together with all result files or outputs of the used analysis software; corresponding scan number can be found in the second column. z = charge: m = mass.

Supplementary Methods

Cross-linking and Tandem Mass Spectrometry - Program Parameters and Settings

For initial cross-link identification, the four different tools were used with the following settings and parameters:

<u>Crux</u>

Crux version 3.0 with the following search string and parameters on single mzML files:
crux search-for-xlinks --enzyme trypsin --fragment-mass mono --precursor-window 5 --precursor-window-type ppm K:E,K:D -18.01056466)

After initial identification, the "xlink-assign-ions" command of Crux was subsequently applied to all detected inter-peptide cross-links between 6xHis-CC1ΔC223 and tubulin with a p-value below 0.05 using the mzML files as input. The "xlink-assign-ions" command, which creates a tab delimited text file with the assigned ions, was applied in the following way:

crux xlink-assign-ions --mz-bin-width 0.02 <peptide A> <peptide B> <pos A> <pos B> -18.01056466 <charge state> <scan number> <mzML file> > "outputfile-name".txt

The inter-peptide cross-links between 6xHis-CC1ΔC223 and tubulin with their assigned ions were considered for final evaluation. Spectra were visualized and annotated using mMass (ver. 5.5.0).

<u>Spectrum Identification Machine (SIM-XL)</u>

SIM-XL version 1.2.2.2: Single RAW files were used as input. A zero-length cross-linker with a mass shift of -18.0106 and reaction sites between E,K and D,K was defined. The precursor mass error was set to 5 ppm, the fragment ion mass error was set to 20 ppm. Trypsin with full specificity was set as the digestive enzyme. The fragmentation method was set to HCD and a variable oxidation of methionine with a mass shift of 15.9549 was considered. Four isotopic possibilities were allowed. Peptides with a minimum of 4 residues per chain, a maximum of 3 missed cleavages, a minimum mass of 600 Da and a maximum mass of 6,000 Da were defined to be analyzed. All detected inter-peptide cross-links between 6xHis-CC1ΔC223 and tubulin were considered for final evaluation.

pLink

pLink version 1.23 was used with an ini-file containing the following parameters on single mgf files:

sample.num=1
sample1.spectra.instrument=HCD
sample1.spectra.format=mgf
enzyme.name=Trypsin
mod.fixed.total=0
mod.variable.total=1
mod.variable.1=Oxidation[M]
linker.total=2
linker.name1=EDC-D
linker.name2=EDC-E
noninterexport=false
drawpsm=false
peptide_tol_total=1
peptide_tol1=10
peptide_tol_type1=ppm
peptide_tol_base1=0.000000
peptide_tol_base_type1=Da
peptide_tol2=10
peptide_tol_type2=ppm
peptide_tol_base2=1.007825035
peptide_tol_base_type2=Da
peptide_tol3=10
peptide_tol_type3=ppm
peptide_tol_base3=2.01565007
peptide_tol_base_type3=Da
peptide_tol4=10
peptide_tol_type4=ppm
peptide_tol_base4=3.023475105
peptide_tol_base_type4=Da
peptide_tol5=10
peptide_tol_type5=ppm
peptide_tol_base5=4.03130014
peptide_tol_base_type5=Da
filter_peptide_tol_base=0,1.007825035,2.01565007,3.023475105,4.03130014
filter_peptide_tol_lb=-10,-10,-10,-10,-10

filter_peptide_tol_ub=10,10,10,10,10
filter_peptide_tol_type=ppm
evalue_max=1
SeparateTypeFilter=1

All detected inter-peptide cross-links between 6xHis-CC1ΔC223 and tubulin were considered for final evaluation.

StavroX

StavroX version 3.6.0.1: Merged mgf files were used as input. Protease sites were set to be R or K and to be blocked by a following P. Maximum number of missed cleavages was set to 3. Oxidation of methionine was defined as a variable modification (max. number=2). EDC was chosen as the cross-linking agent. The precursor mass error was set to 5 ppm, the fragment ion mass error was set to 10 ppm. HCD was defined as the fragmentation method and de-isotoping was enabled. Peptides with a minimum mass of 600 Da and a maximum mass of 6,000 Da were defined to be analyzed. The minimal signal to noise ratio was set to 1.5. Decoy analysis was enabled with standard scoring, an FDR cutoff of 5 % and mixed target decoys. For the decoy analysis the sequences were shuffled but protease sites were kept. All detected inter-peptide cross-links between 6xHis-CC1ΔC223 and tubulin with an FDR<5 % were considered for final evaluation.
