# Supplementary material for: The companion of cellulose synthase 1 confers salt tolerance through a Tau-like mechanism in plants
Source: Nat Commun. 2019 Feb 20;10:857. doi: 10.1038/s41467-019-08780-3 (PMC6382854; doi:10.1038/s41467-019-08780-3)
Supplement: Supplementary file 3 — Description of Additional Supplementary Files [file 41467_2019_8780_MOESM3_ESM.pdf]

Description of Additional Supplementary Files

File Name: Supplementary Movie 1
Description: CF488A-labeled His-CC1ΔC223 proteins (green) diffuse on surfacebound microtubules (magenta) in vitro. Scale bar = 5 µm.

File Name: Supplementary Movie 2
Description: Like GFP-CC1, GFP-CC1YYAA migrates as foci at the plasma membrane. Scale bar = 5 µm.
