# Supplementary material for: The companion of cellulose synthase 1 confers salt tolerance through a Tau-like mechanism in plants
Source: Nat Commun. 2019 Feb 20;10:857. doi: 10.1038/s41467-019-08780-3 (PMC6382854; doi:10.1038/s41467-019-08780-3)
Supplement: Supplementary file 6 — Reporting Summary [file 41467_2019_8780_MOESM6_ESM.pdf]

nature research

Corresponding author(s): Staffan Persson, Hartmut Oschkinat

Reporting Summary

Nature Research wishes to improve the reproducibility of the work that we publish. This form provides structure for consistency and transparency in reporting. For further information on Nature Research policies, see Authors & Referees and the Editorial Policy Checklist.

Statistical parameters

When statistical analyses are reported, confirm that the following items are present in the relevant location (e.g. figure legend, table legend, main text, or Methods section).

n/a	Confirmed	
☐	☒	The exact sample size (n) for each experimental group/condition, given as a discrete number and unit of measurement
☐	☒	An indication of whether measurements were taken from distinct samples or whether the same sample was measured repeatedly
☐	☒	The statistical test(s) used AND whether they are one- or two-sided<br>*Only common tests should be described solely by name; describe more complex techniques in the Methods section.*
☒	☐	A description of all covariates tested
☒	☐	A description of any assumptions or corrections, such as tests of normality and adjustment for multiple comparisons
☐	☒	A full description of the statistics including central tendency (e.g. means) or other basic estimates (e.g. regression coefficient) AND variation (e.g. standard deviation) or associated estimates of uncertainty (e.g. confidence intervals)
☒	☐	For null hypothesis testing, the test statistic (e.g. F, t, r) with confidence intervals, effect sizes, degrees of freedom and P value noted<br>*Give P values as exact values whenever suitable.*
☒	☐	For Bayesian analysis, information on the choice of priors and Markov chain Monte Carlo settings
☒	☐	For hierarchical and complex designs, identification of the appropriate level for tests and full reporting of outcomes
☐	☒	Estimates of effect sizes (e.g. Cohen's d, Pearson's r), indicating how they were calculated
☐	☒	Clearly defined error bars<br>*State explicitly what error bars represent (e.g. SD, SE, CI)*

Our web collection on statistics for biologists may be useful.

Software and code

Policy information about availability of computer code

Data collection	No previously unreported custom computer code or algorithm were used.
Data analysis	FIESTA v1.05.0005, Fiji (multiple versions), Crux v3.0, SIM-XL v1.2.2.2, pLink v1.23,StavroX v3.6.0.1, GUSSI v.142, CCPN analysis v2.4.2, GraphPad Prism 7.01, TAIR (https://www.arabidopsis.org), Sedfit v1501b, SEDNTERP v20130813, Bruker TopSpin software v3.1 and 3.2, Thermo Scientific Orbitrap Tribrid MS Series ICSW and Xcalibur (multiple versions), VisiView (multiple versions, Visitron, Munich, Germany).

For manuscripts utilizing custom algorithms or software that are central to the research but not yet described in published literature, software must be made available to editors/reviewers upon request. We strongly encourage code deposition in a community repository (e.g. GitHub). See the Nature Research guidelines for submitting code & software for further information.

Data

Policy information about availability of data

All manuscripts must include a data availability statement. This statement should provide the following information, where applicable:

- Accession codes, unique identifiers, or web links for publicly available datasets
- A list of figures that have associated raw data
- A description of any restrictions on data availability

The mass spectrometry dataset generated and analyzed during this study are available in the PRIDE Archive with the identifier (PXD009260). The NMR chemical shift data is deposited in the BMRB database with accession number 27660. All other relevant data supporting the findings of this study are available within the article, its Supplementary Information File, and Source data File or upon request from the corresponding authors.

Field-specific reporting

Please select the best fit for your research. If you are not sure, read the appropriate sections before making your selection.

☒ Life sciences ☐ Behavioural & social sciences ☐ Ecological, evolutionary & environmental sciences

For a reference copy of the document with all sections, see nature.com/authors/policies/ReportingSummary-flat.pdf

Life sciences study design

All studies must disclose on these points even when the disclosure is negative.

Sample size	There was no specific statistical method used to determine sample size. However, sample size was chosen according to previous studies (Endler et al., Cell 162: 1353–1364,2015; Paredez et al., Science 312: 1491-1495, 2006; Reuther et al., Nature Nanotechnology 11: 914–915 (2016); Mitra et al., PNAS 115 (34): E7950-E7959.
Data exclusions	No samples were excluded, except when samples were moving during image acquisition and this could not be corrected.
Replication	All experiments were at least repeated three times with multiple biological replicates and independently from each other. All replicates are included in this study.
Randomization	Plants were always randomly distributed in the growth/treatment chambers. Otherwise, no randomization was necessary for this study.
Blinding	Data analysis (especially for images) was either done automatically, so independently from the investigator or file names (e.g. including an indication for genotype or replicate number) were removed for analysis. For the measurement of plant size, investigators were not blinded since this is not relevant to the study. In this case, data were always collected according to the genotype of plants.

Reporting for specific materials, systems and methods

Materials & experimental systems

n/a	Involved in the study
☐	☒ Unique biological materials
☐	☒ Antibodies
☒	☐ Eukaryotic cell lines
☒	☐ Palaeontology
☒	☐ Animals and other organisms
☒	☐ Human research participants

Methods

n/a	Involved in the study
☒	☐ ChIP-seq
☒	☐ Flow cytometry
☒	☐ MRI-based neuroimaging

Unique biological materials

Policy information about availability of materials

Obtaining unique materials	All unique materials used are available from the authors of from standard commercial sources.

Antibodies

Antibodies used	Sigma Monoclonal Anti-ß-Tubulin (#T7816).
Validation	The same antibody was used in multiple studies for the same purpose (e.g. Endler et al., Cell 162: 1353–1364,2015; Gell et al., Methods Cell Bio. 95: 221-245, 2010.)
